# Supplementary material for: Cell-type specific sensory and motor activity in the cuneiform nucleus and pedunculopontine nucleus in mice
Source: Sci Rep. 2025 Jul 1;15:20408. doi: 10.1038/s41598-025-05572-2 (PMC12217477; doi:10.1038/s41598-025-05572-2)
Supplement: Supplementary file 1 — Supplementary Material 1 [file 41598_2025_5572_MOESM1_ESM.pdf]

## SUPPLEMENTARY INFORMATION

**TITLE.** Cell-type specific sensory and motor activity in the cuneiform nucleus and pedunculopontine nucleus in mice.

**AUTHOR LIST.** Cornelis Immanuel van der Zouwen[1], Andrea Juárez Tello[1+], Jacinthlyn Sylvia Suresh[1+], Juan Duque-Yate[1], Ted Hsu[2], Vaibhav Konanur[2], Joël Boutin[1], Mitchell F. Roitman[2], Dimitri Ryczko[1,3,4,5*].

**AFFILIATIONS.**

[1]Département de Pharmacologie-Physiologie, Faculté de médecine et des sciences de la santé, Université de Sherbrooke, Sherbrooke, QC, Canada.

[2]Department of Psychology, University of Illinois at Chicago, Chicago, IL, USA.

[3]Centre de recherche du Centre Hospitalier Universitaire de Sherbrooke, Sherbrooke, QC, Canada.

[4]Neurosciences Sherbrooke, Sherbrooke, QC, Canada.

[5]Institut de Pharmacologie de Sherbrooke, Sherbrooke, QC, Canada.

[+]These authors contributed equally.

*****Correspondence: dimitri.ryczko@gmail.com

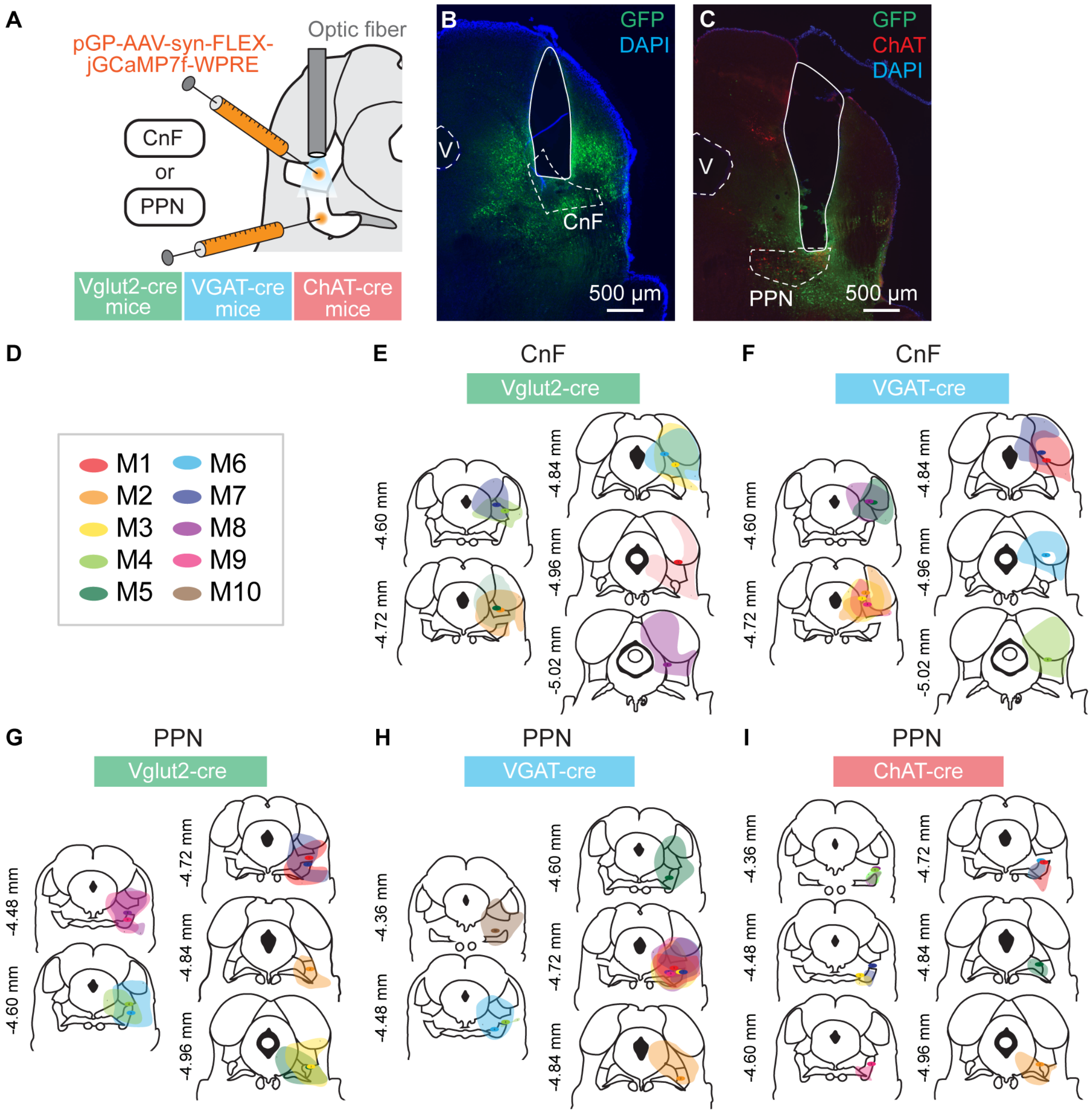


**Figure S1. Adeno-associated virus injections and photometry fiber implantations. (A)** Vglut2-cre, VGAT-cre or ChAT-cre mice were injected in the cuneiform nucleus (**CnF**) or pedunculopontine nucleus (**PPN**) with an adeno-associated virus (**AAV**) encoding for the genetically encoded calcium indicator jGCaMP7f in a cre-dependent manner (see Methods) and implanted with an optic fiber ~150 µm above the injection site. **(B,C)** Photomicrographs showing the position of cells infected by virus injection (green) in CnF or PPN and the position of the optic fiber right above the CnF (B) or PPN (C) in example Vglut2-cre mice, with the nuclear marker DAPI shown in blue. In C, cells immunoreactive for choline acetyltransferase (**ChAT**) are shown in red. **(D)** Color code used to distinguish individual mice (M). **(E-I)** Histological locations of the cells infected by the AAV and of the tips of optic fibers.

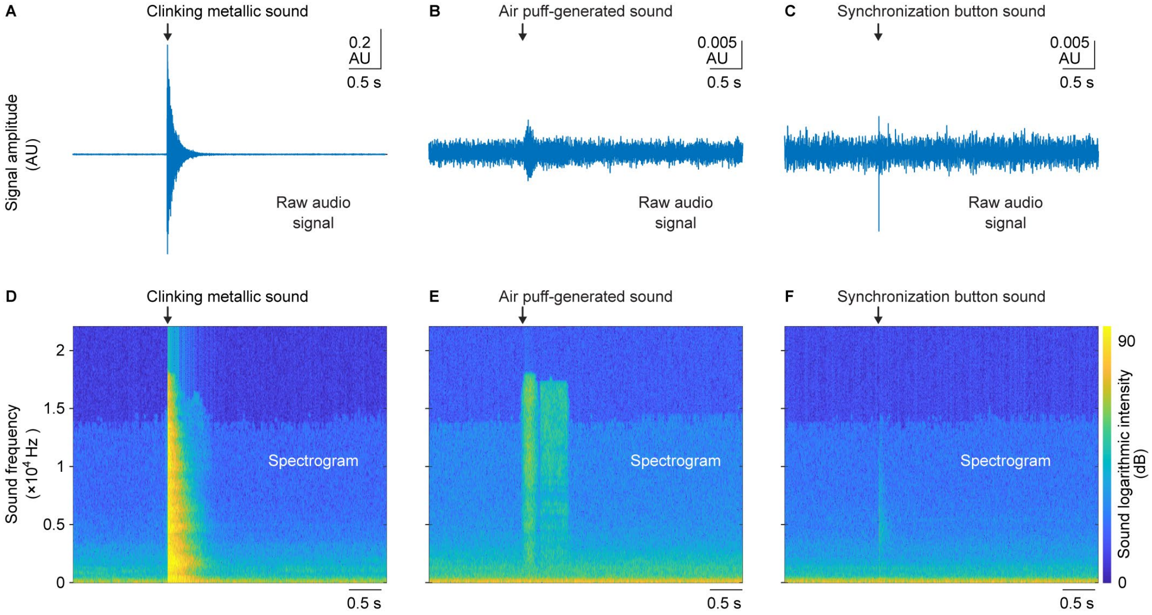


**Figure S2. Sound spectrograms. (A-C)** Raw audio signal for a clinking metallic sound (A), air puff- generated sound (B), and for the sound generated by the button press used to synchronize sensory stimulation and fiber photometry recordings (C). These signals were used to generated spectrograms in D-F (see Methods). **(D-F)** Typical spectrograms obtained for a clinking metallic sound (D), air puff-generated sound (E), and button press sound (F). The x-axis represents time, with a scale similar to that used for raw audio signals in (A-C). The y-axis represents sound frequencies, and the color scale indicates the logarithmic intensity of the sound.

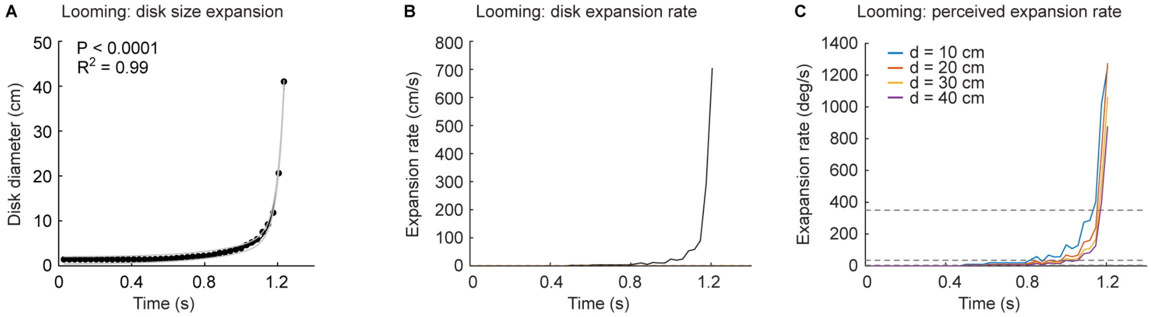


**Figure S3. Visual looming stimulus properties. (A)** Black disk diameter grew exponentially as a function of time throughout the duration of the visual looming stimulus (~1.7 s). Note that beyond 1.2 s, part of the disk exceeds the screen size, making its diameter unmeasurable. The exponential fit's squared correlation coefficient ($R^2$) and significance (P) are provided. **(B)** The disk expansion rate (cm/s) represents the change in disk diameter over time. **(C)** The perceived expansion rate (degrees/s) estimates the rate of change in the diameter of the disk over time, for observers placed either 10 cm (blue line), 20 cm (red line), 30 cm (yellow line) or 40 cm (purple line) away from the screen. The two horizontal dotted lines illustrate the range of expansion rates (35–350 degrees/s) previously determined to evoke locomotor responses [S1].

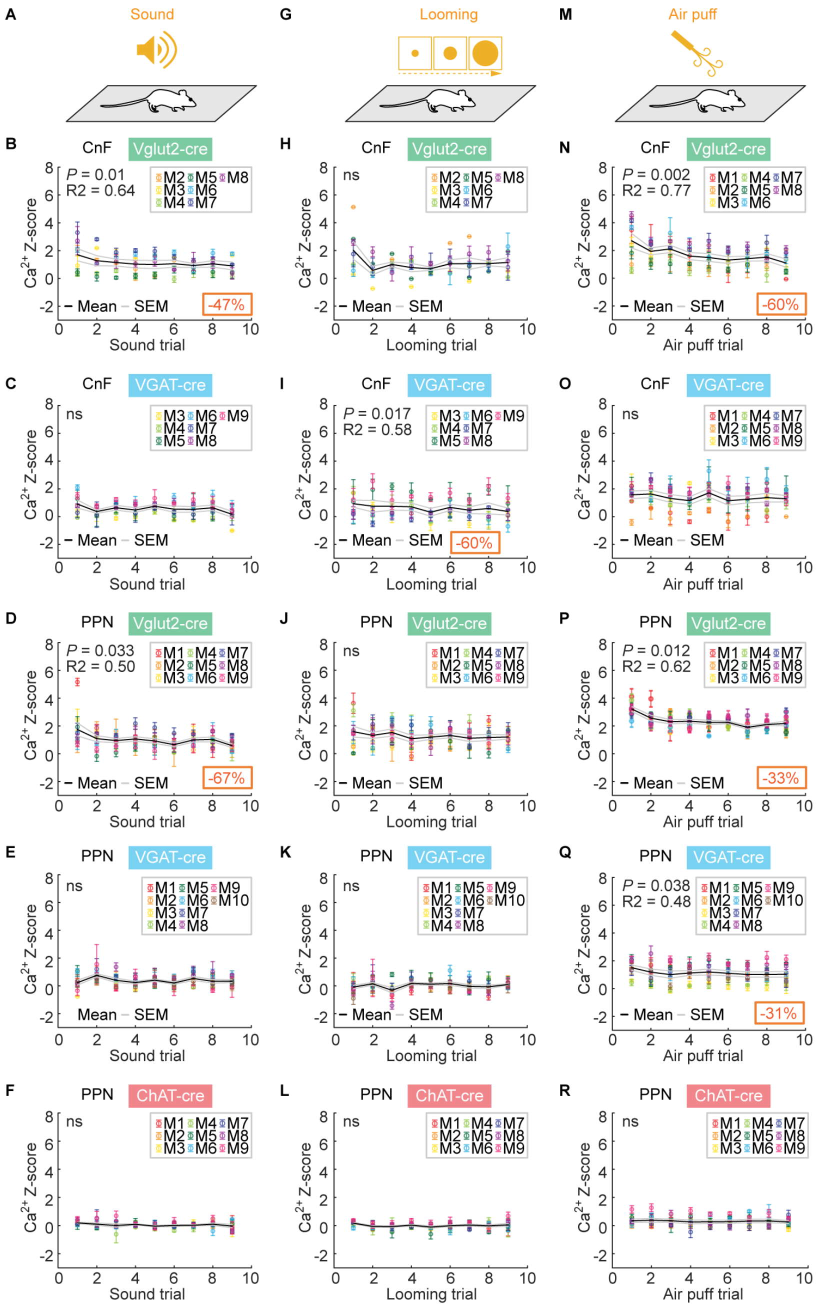


**Figure S4. Habituation to sensory stimulations in CnF or PPN cells. (A-R)** Mean calcium ($Ca^{2+}$) responses recorded in the cuneiform nucleus (**CnF**) or pedunculopontine nucleus (**PPN**) of Vglut2-cre, VGAT-cre or ChAT-cre mice during the first 9 trials of 1-4 series of sound stimulations (A-F), visual looming stimulations (G-L), or air puff stimulations (M-R). For each trial number, the mean $Ca^{2+}$ response ± SEM is illustrated. In each case, the existence of a linear relationship between trial number and $Ca^{2+}$ signal amplitude was tested. When a fit is significant, the coefficient of correlation (R) and its significance (P) are illustrated, as well as the mean percentage decrease of the response from first to last trial (value in orange square) (ns, not significant, linear fit).

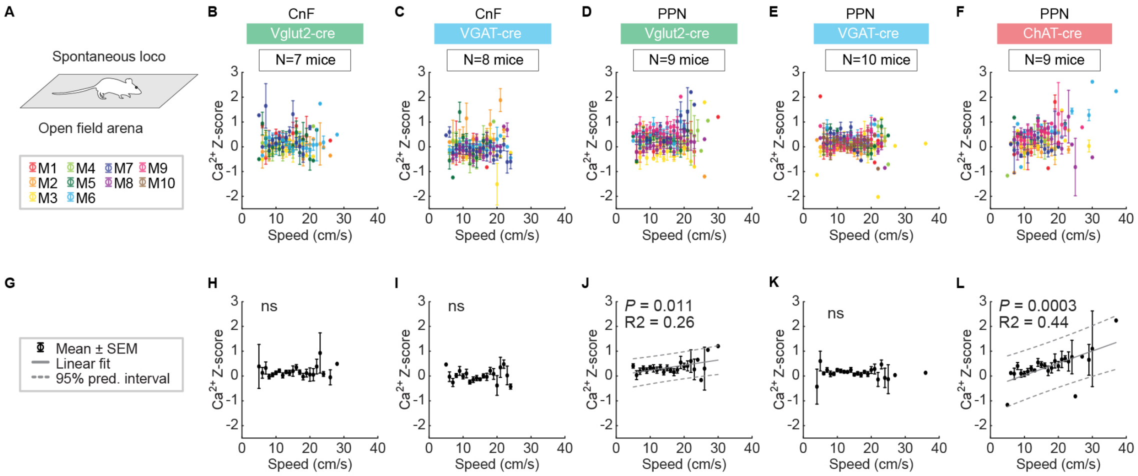


**Figure S5. Correlation between CnF or PPN neuronal activity and movement speed. (A-L)** Mean $Ca^{2+}$ signals in relation to the mean speed per locomotor bout recorded in the CnF or PPN of Vglut2-cre, VGAT-cre or ChAT-cre mice during spontaneous activity in the open field. Locomotor bouts were divided into speed bins of 1 cm/s (A-F) Each colored dot represents the mean $Ca^{2+}$ signal (±SEM) per animal at the corresponding speed, with 1-45 bouts per bin. (G-L) Each dot represents the mean $Ca^{2+}$ signal (±SEM) of 1-10 animals at the corresponding speed. In each case, the existence of a linear relationship between $Ca^{2+}$ signal amplitude and speed was tested. When a fit is significant, the squared coefficient of correlation ($R^2$) and its significance (*P*) are illustrated, as well as the linear fit (solid grey line) and the 95% prediction interval (dotted grey lines) (ns, not significant, linear fit).

## SUPPLEMENTARY REFERENCES

[S1]. Yilmaz, M., and Meister, M. (2013). Rapid Innate Defensive Responses of Mice to Looming Visual Stimuli. Current Biology *23*, 2011–2015. https://doi.org/10.1016/j.cub.2013.08.015.
